# Dietary supplementation of cystinotic mice by lysine inhibits the megalin pathway and decreases kidney cystine content

**DOI:** 10.1038/s41598-023-43105-x

**Published:** 2023-10-12

**Authors:** L. R. Rega, V. Janssens, J. H. Graversen, S. K. Moestrup, S. Cairoli, B. M. Goffredo, N. Nevo, G. E. Courtoy, F. Jouret, C. Antignac, F. Emma, C. E. Pierreux, P. J. Courtoy

**Affiliations:** 1 Nephrology Research Unit, Translational Pediatrics and Clinical Genetics Research Area, Bambino Gesù Children’s Hospital, IRCCS, Rome, Italy; 2 Cell Biology Unit, de Duve Institute and Louvain University Medical School, Brussels, Belgium; 3 Department of Molecular Medicine, University of Southern Denmark, Odense, Denmark; 4 Division of Metabolic Diseases and Drug Biology, Bambino Gesù Children’s Hospital, IRCCS, Rome, Italy; 5 Laboratoire des Maladies Rénales Héréditaires, Inserm UMR 1163, Institut Imagine, Université Paris Cité, Paris, France; 6 Imaging Platform (2IP), Institut de Recherche Expérimentale et Clinique, Louvain University Medical School, Brussels, Belgium; 7 Groupe Interdisciplinaire de Génoprotéomique Appliquée (GIGA), Cardiovascular Sciences, University of Liège, Liège, Belgium

**Keywords:** Endocytosis, Kidney, Fanconi syndrome

## Abstract

Megalin/LRP2 is a major receptor supporting apical endocytosis in kidney proximal tubular cells. We have previously reported that kidney-specific perinatal ablation of the megalin gene in cystinotic mice, a model of nephropathic cystinosis, essentially blocks renal cystine accumulation and partially preserves kidney tissue integrity. Here, we examined whether inhibition of the megalin pathway in adult cystinotic mice by dietary supplementation (5x-fold *vs* control regular diet) with the dibasic amino-acids (dAAs), lysine or arginine, both of which are used to treat patients with other rare metabolic disorders, could also decrease renal cystine accumulation and protect cystinotic kidneys. Using surface plasmon resonance, we first showed that both dAAs compete for protein ligand binding to immobilized megalin in a concentration-dependent manner, with identical inhibition curves by l- and d-stereoisomers. In cystinotic mice, 2-month diets with 5x-l-lysine and 5x-l-arginine were overall well tolerated, while 5x-d-lysine induced strong polyuria but no weight loss. All diets induced a marked increase of dAA urinary excretion, most prominent under 5x-d-lysine, without sign of kidney insufficiency. Renal cystine accumulation was slowed down approx. twofold by L-dAAs, and totally suppressed by d-lysine. We conclude that prolonged dietary manipulation of the megalin pathway in kidneys is feasible, tolerable and can be effective in vivo.

## Introduction

Megalin is a transmembrane protein with a huge extracellular domain which, together with the cubilin:aminonless complex, drives receptor-mediated endocytosis at the apical surface of multiple epithelia for a wide variety of proteins, ions and drugs^1^. Megalin serves as paradigmatic component of apical endocytotic machineries in differentiated cells^2,3^ and during development^4^ and its cargoes are *bona fide* tracers of apical PT function^5^. The resulting flux of apical cargoes and rapid megalin recycling in kidney proximal tubular cells (PTCs) lead to virtually complete reabsorption of ultrafiltrated plasma low-molecular-weight proteins (LMWPs) during the < 1 min tubular transit^6^. The megalin pathway in PTCs thereby supports effective retrieval of several vitamins by mediating uptake of corresponding vitamin:carrier proteins, contributes to lysosome biogenesis^7^ and protease clearance^8^ but is also responsible for gentamicin nephrotoxicity^9^.

In a landmark paper, Mogensen and Sølling reported inhibition of kidney tubular reabsorption of LMWPs by intravenous injection of small cationic compounds in humans^10^. Among these, l-lysine most effectively induced dose-dependent (up to complete) inhibition, with no impact on glomerular filtration. The effect was immediate and reversible, suggesting direct competition for the reuptake machinery, presumably by electrostatic interactions with luminal binding sites. Inhibition of lysozyme recapture by l-lysine and l-arginine was confirmed in microperfusion experiments^11^. Moreover, clinical and molecular studies disclosed the role of megalin for renal uptake of basic compounds and site-directed mutagenesis of cargoes revealed the importance of lysine/arginine residues^9^. These residues interact with Ca^2+^-coordinated clusters of acidic residues in ligand-binding repeats of LDL-receptor family proteins, including megalin^12^.

However, l-lysine gavage to rats for one day, which also triggered prominent LMWPuria, resulted in a striking relocation of megalin and cubilin from the base of the brush border, where endocytosis and receptor recycling take place, to brush border itself, devoid of endocytic vesicles^13^. This suggested freezing of megalin trafficking in otherwise intact PTCs.

Whereas decades of research failed to achieve clinically acceptable competition for gentamicin PTC recapture to prevent its nephrotoxicity, acute inhibition of PTC reuptake of radiopeptides used for cancer therapy by concomitant injection of cationic, dibasic amino-acids (dAAs) is effective to harness radiation-induced nephrotoxicity^14^. This effect depends on the megalin pathway, as demonstrated in cultured cells^15^. On these bases, we hypothesized that targeting the megalin pathway by prolonged dietary supplementation with lysine or arginine, both used to treat rare metabolic disorders^16,17^, could prevent PTC reuptake of noxious proteinaceous megalin cargoes. We here selected the case of nephropathic cystinosis (NC, in brief cystinosis), for which adequate rodent models are available^18–21^ and a role of endocytosis in disease progression has been advocated^22^.

Cystinosis is a rare lysosomal storage disease due to defective *CTNS* gene, encoding for *CYSTINOSIN*, a proton-driven lysosomal membrane cystine exporter^23–26^. As a result, cystine accumulates in all body cells, eventually forming pathognomonic lysosomal crystals. Cystine generation in lysosomes requires cysteine import, since knocking-out the lysosomal cysteine membrane transporter, *MFSD12*, prevents cystine accumulation in cystinotic cells^27^. However, cystine accumulation in cultured cystinotic cells increases with extracellular concentration of disulfide-rich proteins but not their reduced equivalents^28^, suggesting that their disulfide bridges serve during lysosomal processing as oxidative stoechiometric equivalents to convert two imported cysteine molecules into one cystine^29^. The first clinical manifestation of cystinosis is a generalized urinary loss of solutes and LMWPs, named renal Fanconi syndrome. The only current FDA-approved medical treatment of cystinosis is cysteamine, which significantly delays disease progression but is not a cure, calling for novel therapies^30^.

In PTCs, megalin expression and endolysosomal activity naturally decline with cystinosis progression, as part of apical dedifferentiation^31–33^. We however reported that, in *CTNS* knock-out C57bl-6 mice (briefly *Ctns* KO), perinatal kidney-specific genetic ablation of megalin prevents kidney cystine accumulation and mitigates kidney tissue remodeling^22^. We thus suggested that endocytic supply to kidney PTCs of ultrafiltrated, disulfide-rich plasma proteins is an important factor of cystinosis progression, and contemplated the megalin pathway as potential therapeutical target. We here tested in the same *Ctns* KO mice whether diets supplemented with the dAAs, l-arginine or l-lysine, could inhibit kidney cystine accumulation and hopefully offer tissue protection, translatable to cystinotic patients. We also compared l- with d-lysine to discriminate luminal *vs* intracellular/metabolic effects. Indeed, l-lysine is a main cargo of the stereospecific heterodimeric rBAT/b^0,+^AT complex (SLC3A1/SLC7A9) which drives its apical reabsorption^34^. Conversely, d-lysine should be reabsorbed during its slow progression across the digestive tract, but not appreciably transported into PTCs during the extremely rapid urine passage. A recent paper revealed the importance of lysine metabolism in kidney cortex, its alteration in a rat model of sodium-induced hypertension with kidney damage, as well as the clinical, structural, and metabolic benefit of dietary supplementation by l-lysine in this model^35^.

In this report, we integrate information on the effects of lysine diet on megalin/PTCs based on four different approaches : (i) *acellular* competition on chip-immobilized megalin biosensor; (ii) 5x-l-lysine diet in wild-type (WT) *mice* on megalin localization and endocytic tracing in kidney cortical PTCs; and diet effects in *Ctns* KO mice kidneys for either (iii) primary prevention (2-to-6 months of age; to best mimic perinatal genetic ablation), or (iv) secondary treatment (6-to-8 months of age, in translational perspective, the main goal of this paper). 5x-l-Arginine and 5x-d-lysine diets will be tested for comparison.

## Results

### Effect of dibasic amino-acids on megalin ligand binding in acellular conditions

**Figure 1:**
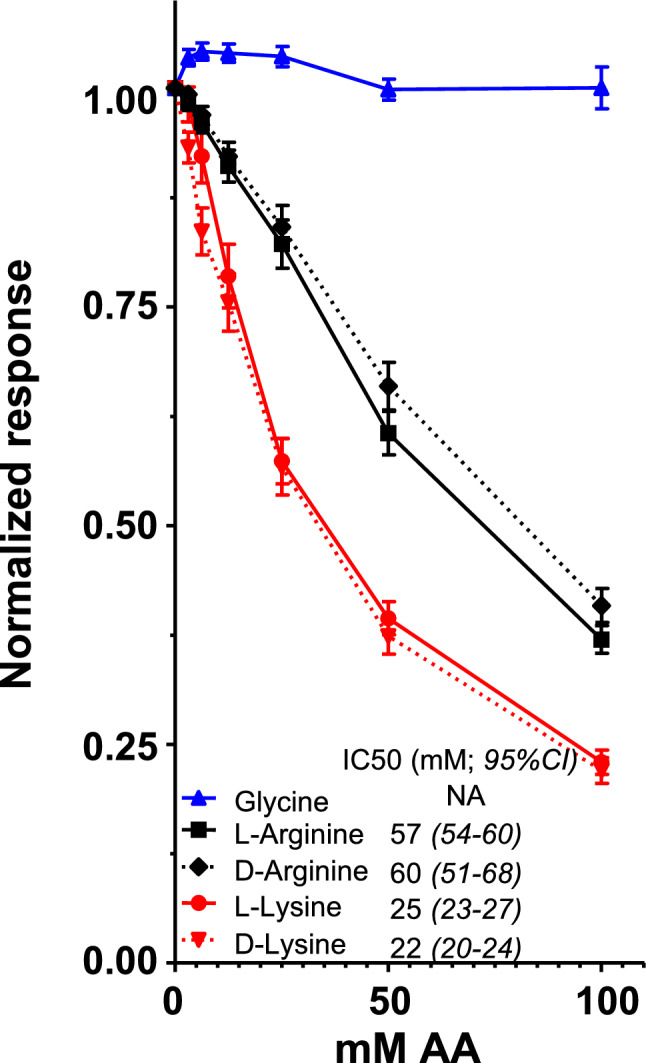
Acellular assay: direct competition for transcobalamine:cobalamine binding to megalin immobilized on Biacore sensor chips. Signals of sensorgrams (shown in Supplemental Fig. 1) were normalized to control signal without AA supplementation, taken as the unity, and are means ± SEM of at least four independent technical duplicate values at each of the indicated amino-acid-concentrations. Notice (i) no effect of chiral glycine; (ii) superimposable inhibitory curves for each L-/D-AA pair; and (iii) approx. twofold higher potency of lysine *vs* arginine in this assay.

To directly address whether supra-physiological concentrations of dibasic amino-acids (dAAs) can interfere with binding of protein ligands to megalin, we used surface plasmon resonance. Sensor chips were derivatized with porcine megalin at high density. When chips were exposed to increasing concentrations of non-chiral glycine as control, of l- *vs* d-arginine, or l- *vs* d-lysine, no signal was detected. After flushing, chips were incubated with 10 µg/ml of three analytes: the megalin chaperone, RAP (receptor-associated protein), as positive control (not shown), or two physiological megalin ligands: transcobalamine:cobalamine complexes (TC:B12; Fig. 1; sensorgrams shown at Supplemental Fig. 1a) or lysozyme (Supplemental Fig. 1b), diluted in 0 to 100 mM of the same amino-acids in buffer. No detectable effect up to 100 mM glycine was observed on megalin:analyte interaction. In contrast, both l- and d-arginine, as well as l- and d-lysine, caused a concentration-dependent decrease of megalin binding of the two natural protein ligands. Remarkably, there was no difference between l- *vs* d stereoisomers of each dAA. IC50 for TC:B12/megalin interaction was ~ 25 mM for lysine *vs* ~ 60 mM for arginine, indicating stronger competition by lysine, consistent with seminal observations of Mogensen and Sølling^10^ and respective IC50 for inhibition by l-lysine *vs* l-arginine of albumin uptake by OK cells^35^. No effect was observed at the physiological concentration of dAAs for rBAT/B^0,+^AT activity (~ 100 µM)^34^, as expected for optimal megalin function. The supraphysiological range for effective competition in vitro defined the necessary increment in urinary concentration from dietary supplementation for significant competition in vivo.

### Pilot testing of prolonged 5x-dibasic amino-acids diet supplementation in WT mice

**Figure 2:**
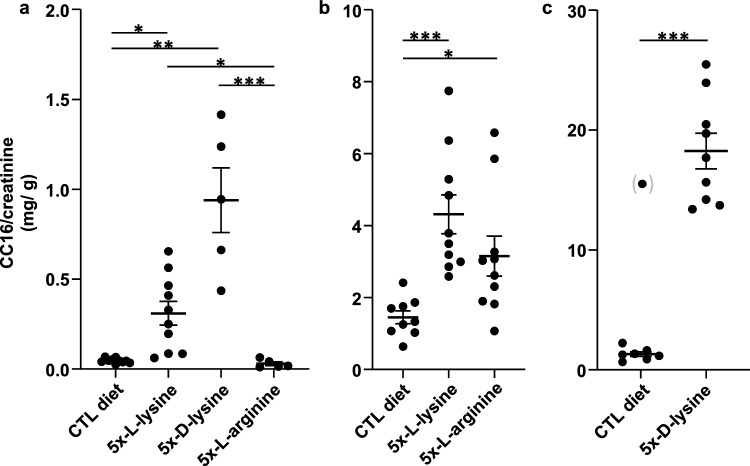
Effects of dAAs on LMW proteinuria in WT and *Ctns* KO mice as monitored by normalized urinary CC16 excretion *(note different scales in the ordinates).* (**a**) Wild-type mice, 3 weeks of diet. (**b**) *Ctns* KO mice, first study, comparison between 5x-l-lysine with 5x-l-arginine after 2 months of diet (from 6-to-8 months of age). (**c**) *Ctns* KO mice, second study, effect of 5x-d-lysine after 2 months of diet (from 6-to-8 months of age). Statistical significance of differences was determined by Kruskal–Wallis test at (**a**,**b**) and by Mann–Whitney test at (**c**), where one verified outlier under control diet, indicated between parentheses, has been excluded for statistical analysis. *P < 0.05; **P < 0.01; ***P < 0.001.

We next examined the feasibility and tolerance of prolonged dAAs supplementation in mice. As previous reported, *1-day rat* gavage with l-lysine (80 mmoles/kg) is well tolerated, while inducing impressive LMWPuria^13^. We first explored in WT C57Bl-6 *mice* the effect of ad libitum solid diet enriched 5× over regular diet with l-lysine- or l-arginine (45 mmoles/kg/day) for *3 weeks*, by reference to isonitrogenous glycine for control. In a second experiment, 5x-d-lysine was compared to regular diet. As shown by Fig. 2a, 5x-l-lysine and especially 5x-d-lysine markedly increased urinary excretion of CC16 (normalized for creatinine excretion), a sensitive marker of LMWPuria (by 7-fold and 20-fold respectively). In contrast, 5x-l-arginine did not detectably increase CC16 in urine of WT mice.

**Figure 3:**
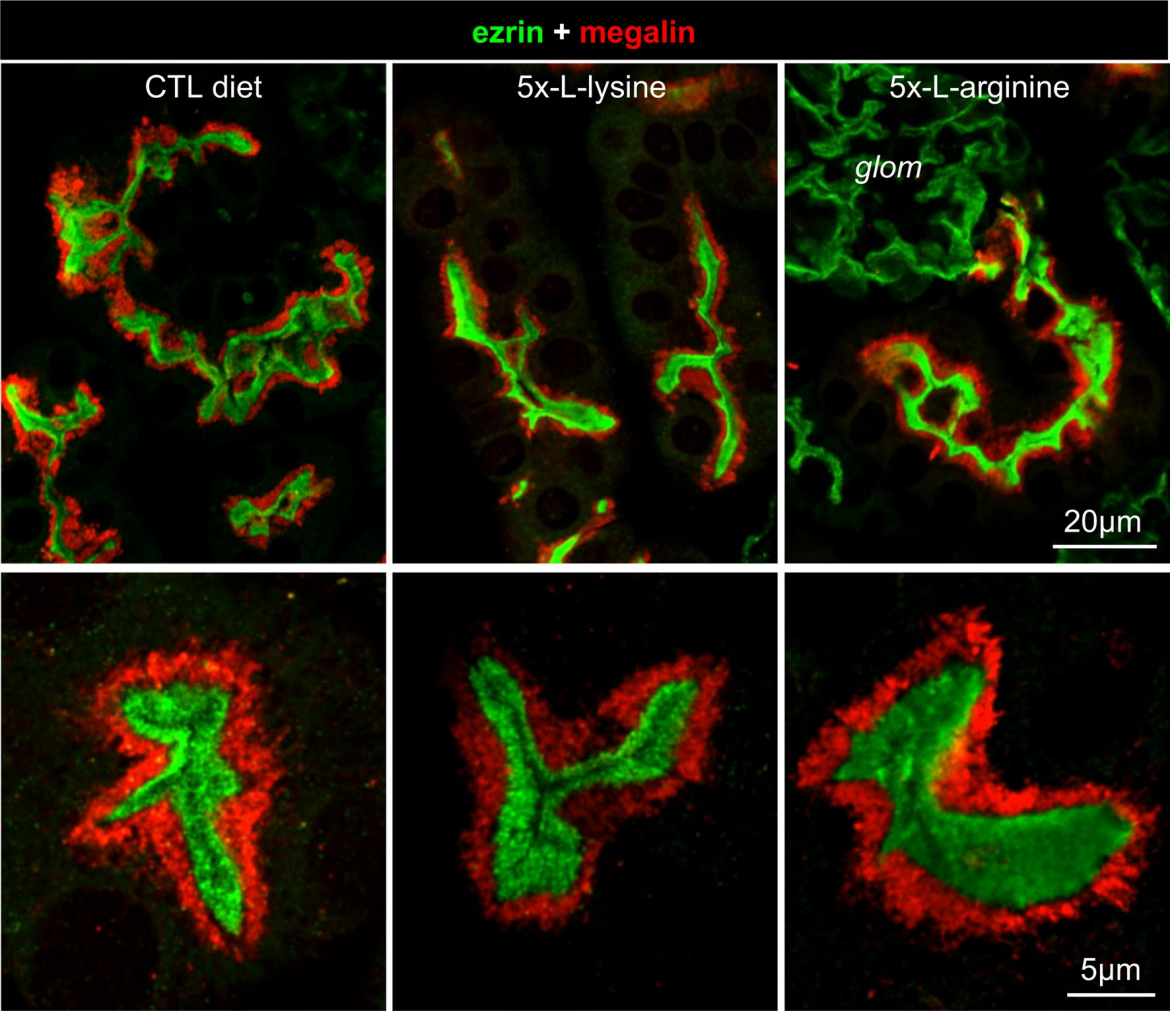
Confocal imaging of megalin in kidney proximal tubular cells of WT mice: no relocation under 5x-l-lysine or 5x-l-arginine. Paraffine sections of kidney cortex from 2 months-old WT C57BL/6 female mice after two weeks under control diet (here, isonitrogenous glycine), 5x-l-lysine or 5x-l-arginine, were immunolabeled for megalin (red) by reference to brush border (ezrin, green). At low magnification (upper panel; scale bar 20 µm) subcellular localization of the major megalin pool remains as a subapical layer in all conditions. At higher magnification (lower panel; scale bar, 5 µm), the megalin layer is sharply separated from the green ezrin layer by a dark thin intermediate layer, without any overlap, thus does not appear to be mislocalized by any of the diets. *Glom*, glomerulus (no detectable megalin signal).

However, 1-day l-lysine gavage in rats also caused a major relocation of the apical endocytic receptors, megalin and cubilin, from the base of brush border (defined by phase contrast) under control diet to its entire thickness, suggesting interference with their endocytic trafficking^13^. In contrast, after 3-weeks 5x-l-lysine diet in WT mice, we found no change of the main megalin pool, remaining immediately *below* the brush border (identified by immunolabelling with ezrin; Fig. 3).

**Figure 4:**
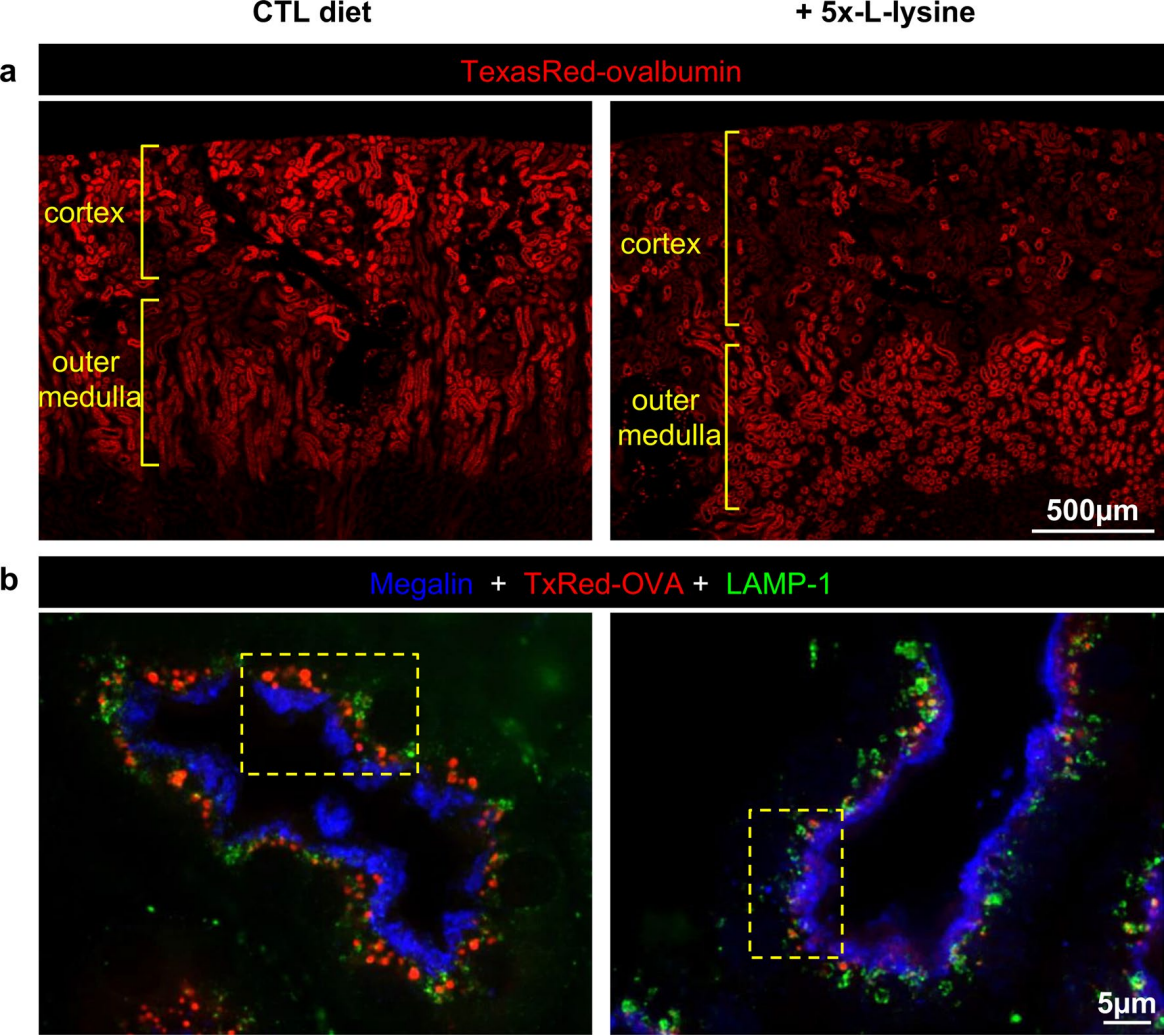
In vivo tracing of acute endocytic protein uptake by kidney proximal tubular cells of WT mice: redistribution from cortex to outer medulla. Two months-old C57BL/6 WT mice were supplemented or not for 3 weeks by 5x-L lysine under inverted 24-h light cycles (initiated 1 week prior diet) to reprogram feeding during day light, so as to maximize plasma thus urine lysine level during endocytic uptake experiment. Twenty min after injection of TexasRed-ovalbumin (TxR-OVA), kidneys were perfusion-fixed. (**a**) Low magnification of kidney cortex and outer medulla (scale bar 500 µm). Under regular diet, acute TxR-OVA uptake is concentrated in kidney cortex (S1/S2 segments). Under 5x-lysine, cortical uptake is damped and tracer is transferred to S3 segment in outer medulla. (**b**) High magnification of cortex after immunolabeling of megalin (blue) and lysosomes (LAMP-1, green) (scale bar 5 µm). Again, 5x-l-lysine does not modify megalin stratified subapical localization. Although red signal is much weaker under l-lysine, contacts of TxRed-OVA-filled late endosomes with lysosomes are preserved.

We also examined whether apical endocytic trafficking of megalin cargo could be blocked under 5x-l-lysine, using apical endocytic tracing in WT mice with the fluorescent megalin ligand, TexasRed-ovalbumin (TxR-OVA). Normally, mice feed at *night* hours. To optimize PT exposure to ingested lysine during endocytic tracing performed at *day* hours, mice were first reprogrammed by adaptation to inverted 24 h-light. As shown by low-magnification at Fig. 4a, TxR-OVA fluorescence intensity in cortical PTCs under 5x-l-lysine-supplemented pellets was greatly decreased during inverted daytime (“reprogrammed feeding hours”), demonstrating inhibition of endocytic capture in vivo. We also noted strong redistribution of the endocytic load from S1/S2 (cortex) to S3 segment (outer medulla), indicating distal compensation. This implied that the extent of competition in cortical PTCs could be greatly underestimated by measured LMWPuria. At higher magnification, contacts of TxR-OVA labeled apical endosomes with lysosomes in cortical PTCs were still visible under 5X-l-Lysine, arguing against a major impairment of apical endosomal trafficking (Fig. 4b). We overall concluded that prolonged l-lysine dietary supplementation can induce LMWPuria without apparently affecting apical megalin trafficking in mice, supporting instead direct in vivo competition for megalin binding during feeding hours. Conversely, we found no or inconsistent S1-to-S3 Tx-OVA redistribution by 5x-Lysine after tracer injection during day hours under normal light-cycle (“starvation hours”), indicating that inhibition is reversible when mice do not feed, thus protection may be discontinuous.

### In vivo tolerance and extent of AAuria after long-term 5x-dibasic amino-acids diet in Ctns KO mice

We next looked whether 5x-l-lysine diet was also well tolerated, effective and potentially protective in *Ctns* KO mice, as first validated rodent model of cystinosis. Since virtually complete perinatal ablation of megalin in these mice largely prevented renal cystine accumulation and attenuated PTC remodeling^22^, we hypothesized that even partial or discontinuous dietary inhibition of this pathway could decrease the uptake of megalin ligands and offer some PTC protection. Since cystinotic patients and mice exhibit muscle defects, l-lysine diet was compared to supplementation by l-arginine, a popular diet to increase muscle mass. We also attempted to further discriminate between luminal (competition for surface megalin binding) and intracellular metabolic effects (see Discussion) by comparing the effects of 5x-l-lysine to its stereoisomer, 5x-d-lysine. Unless stated otherwise, diets were initiated at 6 months-of-age, and mice were studied then euthanized at 8 months-of-age (2-months treatment). This time frame was selected for two reasons. First, in the mouse cystinosis model, this an appropriate window to evaluate the impact on PTC alterations based on changes in kidney cystine accumulation and overall function. At 6 months-of-age, female *Ctns* KO mice have developed a mild Fanconi syndrome, including a 1.5-fold increased diuresis and significant glucosuria as compared to WT age-matched controls, and kidney cystine accumulation increases considerably during the 6-to-8 months interval^18,22,31,32^. Second, although primary prevention by earlier intervention (from 2-to-6 months-of-age) better mimics perinatal genetic megalin ablation, thus could be more efficient, secondary intervention (starting at 6 months-of-age), when disease becomes clinically apparent, is more relevant for further translational studies.

As shown in Table 1 summarizing data on daily urinary collections, L-dAAs diets were well tolerated by *Ctns* KO mice. In contrast to 1-day l-lysine gavage of WT rats^13^, or short-term feeding in a rat model of hypertension^35^, long-term 5x-l-lysine diet from 6-to-8 months-of-age did not durably increase diuresis in *Ctns* KO mice, suggesting adaptation over time. Conversely, 5x-d-lysine caused a *further* doubling of daily diuresis, corresponding to almost one quarter of body weight, whose stability indicated a remarkable capacity to compensate by polydipsia. Urine biochemical analyses normalized to creatinine level (Table 1) indicated that reabsorption of glucose and phosphate was not impaired under 5x-l-lysine or 5x-l-arginine (instead, tended towards lower values). In contrast, glycosuria also doubled under 5x-d-lysine. Phosphaturia showed the same tendency, although without statistical significance. These data pointed to some worsening of the modest Fanconi syndrome in these animals.

**Table 1:** Clinical analyses in *Ctns* KO mice at 8 months of age (2 months of treatment).

	Control	5x-l-arginine	5x-l-lysine	5x-d-lysine
	(n = 16)	(n = 10)	(n = 10)^##^	(n = 9)^###^
Body weight (g)^#^	23.9 ± 0.4	24.9 ± 0.4	23.0 ± 0.9	22.4 ± 0.3
Urine values				
Diuresis (ml/day)	2.4 ± 0.2	2.9 ± 0.3	2.8 ± 0.2	5.3 ± 0.8***
Creatinine (mg/dL)	23.3 ± 2.0	22.1 ± 2.7	25.6 ± 2.5	13.4 ± 1.2**
Daily creatinine output (mg/day)	0.5 ± 0.0	0.6 ± 0.1	0.7 ± 0.1	0.7 ± 0.1
Urea/creatinine (g/g)	177.9 ± 12.2	238.2 ± 17.3	173.0 ± 15.2	181.8 ± 11.6
Glucose/creatinine (g/g)	27.1 ± 4.5	25.8 ± 4.0	19.0 ± 4.6	54.4 ± 4.5**
Phosphate/creatinine (g/g)	3.3 ± 0.6	1.9 ± 0.2	2.3 ± 0.4	4.8 ± 0.4
Microalbumin/creatinine (mg/g)	0.2 ± 0.0	0.2 ± 0.0	0.1 ± 0.0	0.5 ± 0.2** (n = 8)
CC16/creatinine (μg/g)	1378 ± 128.3	3151 ± 556.1*	4314 ± 540.3***	18,257 ± 1488***
Serum values				
Creatinine (mg/dL)	1 ± 0.1	0.9 ± 0.1	0.8 ± 0.1* (n = 9)	0.8 ± 0.1*
Urea (mg/dL)	55.8 ± 5.3	63.6 ± 3.8	58.3 ± 2.6 (n = 9)	43.2 ± 2.0
Cystatin-C (ng/mL)	739.9 ± 38.8	1092 ± 56.5*	669.2 ± 33.2	723.3 ± 32.7
Glucose (mg/dL)	176.2 ± 11.7	170.9 ± 9.5	169.1 ± 9.3 (n = 9)	172.4 ± 7.9
Phosphate (mg/dL)	10.1 ± 0.2	9.7 ± 0.3	10.1 ± 0.5 (n = 9)	9.5 ± 0.4

Data are presented as means ± SEM. *P*-values < 0.05 were considered significant and further defined as follows: *P < 0.05; **P < 0.01; ***P < 0.001. ^#^Average body weight at 6 months of age (T0) was 22.6 g ± 0.3 (n = 45). ^##^n = 10 except for indicated serum values (n = 9); ^###^n = 9 except for indicated microalbuminuria (n = 8). Calculated creatinine clearance (in µL/min) are respectively: control diet, 34.9 ± 2.6 (n = 15); 5x-l-arginine, 40.7 ± 3.5 (n = 8) *NS*; 5x-l-lysine 57.0 ± 7.3* (n = 8); 5x-d-lysine 57.3 ± 7.4* (n = 8).

As deduced from normalized measurements by mass spectrometry of AAs in daily urine collections from *Ctns* KO mice, reported at Table 2, baseline concentrations of arginine (44 µM) and lysine (120 µM) were respectively ~ 1000-fold and ~ 200-fold lower than IC50 determined by Biacore for inhibition of TC/B12 binding to megalin. Under 5x-l-arginine diet, argininuria increased by 33-fold to reach ~ 1 mM, still below IC50 for TC/B12. In parallel, lysinuria and cystinuria increased by eightfold and twofold, respectively, indicating modest cross-competition for the shared rBAT/b^0,+^AT apical exchanger. Under 5x-l-lysine diet, lysinuria increased 210-fold to reach ~ 25 mM, close to its IC50; argininuria and cystinuria increased by 25-fold and 8-fold, respectively, compatible with stronger cross-competition upon lysine diet for rBAT/b^0,+^AT. A fourfold difference of affinity of rBAT/b^0,+^AT between lysine and arginine has been documented in transfected COS-7 cells^36^. By contrast, neither 5x-l-arginine nor 5x-l-lysine affected the concentration of l-glycine and l-alanine, two major neutral AAs in mouse urine. The much lower increase of argininuria under 5x-l-arginine as compared to that of lysinuria under 5x-l-lysine might be explained by active arginine splanchnic metabolism/gut excretion^37^. This interpretation is supported by limited increase of serum arginine upon 5x-l-arginine (137.2 ± 35.0 µM *vs* 108.0 ± 34.5 µM under control diet, n = 4; NS) as compared with serum lysine in mice receiving 5x-l-lysine (580.6 ± 15.5 µM *vs* 298.3 ± 38.8 µM under control diet; n = 4; *P* < 0.05). Replacing 5x-l-lysine by its stereoisomer, 5x-d-lysine, induced a huge urinary excretion of lysine, reaching 2200-fold higher values than under control diet, i.e. ~ 150 mM. Although mass spectrometry cannot discriminate L- from D-AAs, we suspect that most of this increase is due d-lysine which PTCs failed to reabsorb. In parallel, argininuria and cystinuria increased by 12- and 7-fold, respectively. There was no significant change (glycine) or modest increase (alanine) of neutral AAs, indicating essentially preserved reabsorption by PTCs under 5x-d-lysine.

**Table 2:** Effects of diets on urinary AA concentration in *Ctns* KO mice at 8 months of age.

	Control	5x-l-arginine	5x-l-lysine	5x-d-lysine
	(n = 17)	(n = 10)	(n = 10)	(n = 9)
bAA transporter substrates				
Arginine (mM/M creatinine)	22.0 ± 7.3	721.4 ± 196.8***	543.6 ± 99.9***	265 ± 27.5**
Lysine (mM/M creatinine)	56.2 ± 8.5	450.5 ± 147.4	11,820 ± 1932**	123,851 ± 8548***
Cystine (mM/M creatinine)	64.8 ± 26.1	146.6 ± 43.7	504.5 ± 61.2***	481.6 ± 32.8***
Representative neutral AAs				
Glycine (mM/M creatinine)	918.2 ± 196.8	963.7 ± 218.1	881.0 ± 265.9	1066 ± 162
Alanine (mM/M creatinine)	818.8 ± 475.3	712.5 ± 216.9	639.6 ± 279.6	1097 ± 106.5**

Data are from 24-h urine collections and presented as means ± SEM. **P < 0.01; ***P < 0.001.

Serum analyses disclosed no significant increase of urea, creatinine and cystatin-C (except a moderate cystatin-C increase under 5x-l-arginine), indicating preserved glomerular filtration. Due to a systematic decrease of plasma creatinine, calculated creatinine clearance under 5x-l- and d-lysine was *increased* by 1.4-fold, but not under l-arginine (legend of Table 1). Conceivably, this might reflect altered muscle metabolism under lysine supplementation in the cystinosis context, but this remains to be clarified.

### Evaluation of competition for the megalin pathway upon long-term diet supplementation by dibasic amino-acids in Ctns KO mice based on LMWproteinuria

In *Ctns* KO mice kept under control diet till 8 months-of-age, CC16 urinary concentration reached 1387 ± 128.3 µg/g creatinine, a 21.1-fold higher value than in WT mice (65.7 ± 15.8 µg/g), indicating a moderate Fanconi syndrome. CC16 further increased relative to control diet by 1.4-fold under 5x-l-arginine (*P* < 0.05, Fig. 2b), by twofold under 5x-l-lysine (*P* < 0.001, Fig. 2b) and by eightfold under 5x-d-lysine (*P* < 0.001, Fig. 2c). There was no detectable increase of microalbuminuria under 5x-l-arginine nor 5x-l-lysine as compared with values in age-matched *Ctns* KO female mice under control diet, but a > twofold increase under 5x-d-lysine was observed (Table 1 and Supplemental Fig. 2). The increase under d-lysine was qualitatively confirmed by higher intensity of silver-stained bands after SDS-PAGE of creatinine-normalized urine samples, at the position of intact albumin and its major proteolytic products (data not shown). Lack of albuminuria despite disappearance of albumin signal in cystinotic PTC lysosomes upon 5x-l-lysine diet (Supplemental Fig. 3) is compatible with full compensatory distal uptake, as shown by TxRed-OVA tracing in WT kidneys (Fig. 4a).

Altogether, we concluded that prolonged diet supplemented by 5x-l-arginine or 5x-l-lysine were well tolerated by *Ctns* KO mice, increased argininuria and especially lysinuria respectively, and caused a modest enhancement of LMWPuria (presumably underestimating competition in cortical PTCs, see Discussion). By contrast, 5x-d-lysine caused a huge increase of urinary lysine, resulting in more robust increase of urinary excretion of CC16 and increased albuminuria. Thus, the respective order of LMWPuria increase matched the corresponding order of dAA urinary increase. We concluded that 5x-d-lysine resulted in an almost complete competition for megalin binding, although partial interference with the overall PTC function or toxicity cannot be excluded (in view of polyuria and glycosuria).

### Effect of long-term diet supplementation by dibasic amino-acids on kidney cystine accumulation in Ctns KO mice

**Figure 5:**
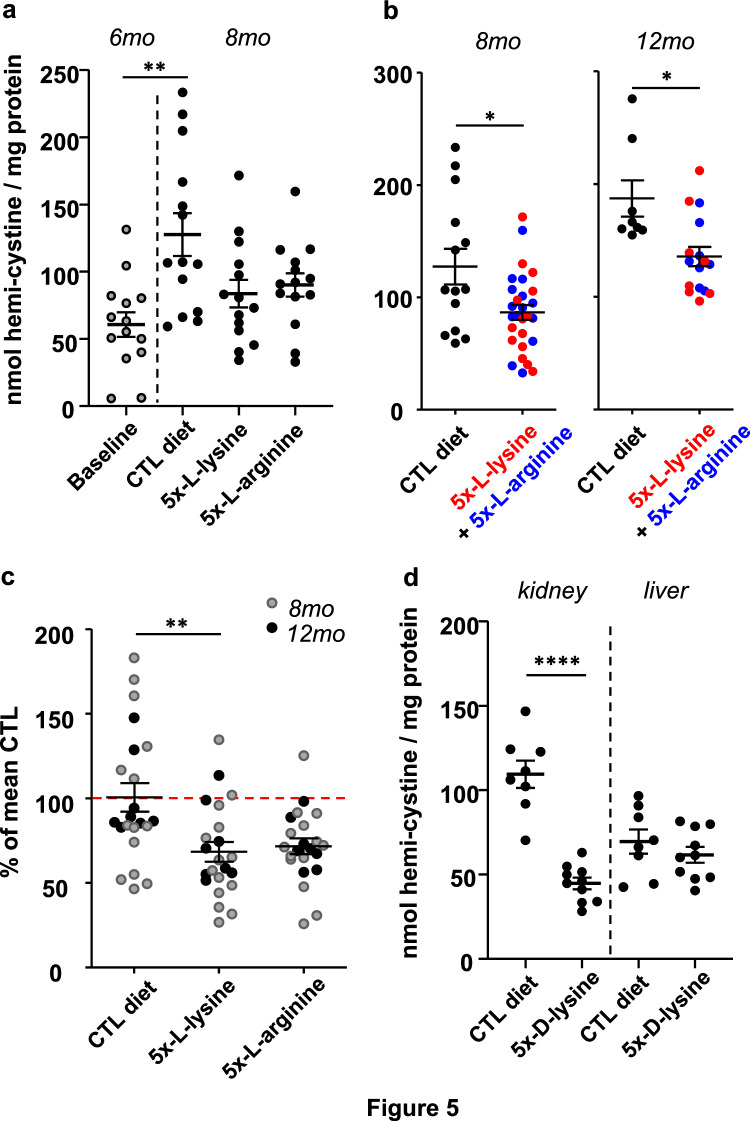
Effect of dAA diets on organ cystine content in *Ctns* KO mice. a-c. Comparison of L-dAA diets on kidney cystine (first study). (**a**) Comparison of absolute values of kidney cystine content normalized to tissue protein at 6 months of age under control diet (baseline) *vs* 8 months of age under the indicated diets. Note that the twofold natural increase of kidney cystine under control diet *(P* = 0.0033) tends to be blunted by both 5x-l-lysine and 5x-l-arginine diets after 2 months of treatment. (**b**) Comparison of normalized absolute kidney content under control diet *vs* combined 5x-l-lysine or 5x-l-arginine diets at 8 months of age (2 months of treatment; *same data as at panel a)* or 12 months of age (6 months of treatment). Comparisons of combined diets at both intervals are statistically significant at *P* < 0.05. (**c**) Comparison of kidney cystine content under individual diet as percentage of mean control diet (red dotted line) for each L-dAA taken separately at combined intervals (*complementary representation of data at panel b*). The effect of l-lysine alone is significant at *P* < 0.01. (**d**) Effect of d-lysine + comparison of organs (second study). Cystine level in kidney *versus* liver in *Ctns* KO mice under control diet or 5x-d-lysine diet from 6-to-8 months of age. Notice full blunting of kidney cystine accumulation under d-lysine, but no effect on liver. Statistical significance of differences was determined by Kruskal–Wallis test at (**a**) and by Mann–Whitney test at (**b**). **, *P* < 0.01; ****, *P* < 0.0001.

For this pilot study, we selected tissue accumulation of cystine as a clear and measurable outcome to quantify long-term interference of dAAs with the endocytic supply of disulfide-rich plasma proteins to kidney PTCs. As shown in Fig. 5a, the mean level of kidney cystine in *Ctns* KO female mice increased under control diet from 60.5 ± 34.3 nmol hemi-cystine/mg kidney protein at 6 months-of-age (baseline) to 127.6 ± 59.3 at 8 months-of-age respectively, thus almost doubled (*P* < 0.005). The mean further accumulation of cystine at 8 months-of-age was similarly reduced in mice under 5x-l-arginine or 5x-l-lysine to reach 90.0 ± 32.3 and 83.6 ± 38.7 nmol hemi-cystine/mg kidney protein, respectively, but did not separately reach statistical significance. However, when two diets are pooled (Fig. 5b, left), the mean kidney cystine in dAA-treated mice at 8 months-of-age, 86.8 ± 35.1 nmol hemi-cystine/mg protein, is significantly lower than under control diet (*P* < 0.05). Of note, the absolute *increment* in kidney cystine from 6-to-8 months of age was attenuated from a value of 67.1 (control diet) down to 26.3 (pooling both dAAs). In other words, the actual *rate* of cystine accumulation over the two-months of treatment period slowed down by ~ 60% under dAA supplementation.

Extending 5x-l-lysine diet over 6 months (till 12 months-of-age) showed persistence of kidney cystine accumulation (Fig. 5b, right). When normalized kidney cystine values under 5x-l-lysine are further expressed as percentages relative to the mean under corresponding control diet and pooled for 8 and 12 months-of-age, they are significantly lower than control (Fig. 5c, *P* < 0.01), i.e. without pooling with 5x-l-arginine data. A similar trend is found for 5x-l-arginine, without reaching statistical significance.

In the second study to explore the effects of the d-lysine stereoisomer, *Ctns* KO mice received control *vs* 5x-d-lysine diet from 6-to-8 months-of-age. Mean kidney cystine value at 8 months-of-age under control diet was 109.4 ± 23.0 nmol hemi-cystine/mg kidney protein, slightly lower than in the first study. In contrast, under 5x-d-lysine, mean kidney cystine value was strongly decreased, to 44.6 ± 10.8 nmol hemi-cystine/mg kidney protein (Fig. 5d, left). This level is very close to baseline at 6 months-of-age (Fig. 5a, left), indicating that kidney cystine accumulation was probably fully arrested under 5x-d-lysine during the treatment period. Of note, d-lysine did not affect liver cystine level at 8 months-of-age, underlining the specificity of kidney effect by this diet (Fig. 5d, right).

### Effect of long-term diet supplementation by l-dibasic amino-acids on kidney histology in Ctns KO mice

We finally evaluated whether decreased cystine accumulation under dAA diets from 6-to-8 months-of-age correlated with significant PTC protection. By histological survey, no obvious change was evidenced after HE or PAS staining (Supplemental Fig. 4a). We thus scored the abundance of characteristic swan-neck lesions at the glomerulo-tubular junction, a quantitative marker disease^22^, and again found no detectable protection (Supplemental Fig. 4b). However, at 6-months-of-age as baseline, most junctions were already altered (~ 80%), leaving little scope for a potential benefit. In contrast, re-examination of a previous cohort targeting primary prevention (supplemented diet from 2-to-6 months-of-age) revealed a remarkable decrease of swan-neck frequency at 6 months-of-age, from ~ 80% under control diet down to ~ 20% under 5x-l-lysine (*P* < 0.01; Supplemental Fig. 5). Taken together, these data suggest that 5x-l-lysine protects PT histology in *Ctns* KO mice if introduced early enough for primary prevention, but offers little advantage by secondary treatment, despite strong decrease of kidney cystine content. To look for fibrosis, histological sections at 8 months-of-age were stained with Sirius-red; its signal was automatically divided into three intensity levels over the entire kidney cortex. This quantification disclosed no detectable decrease of Sirius-red staining, thus fibrosis, upon dAAs at this interval (Supplemental Fig. 4c,d).

**Figure 6:**
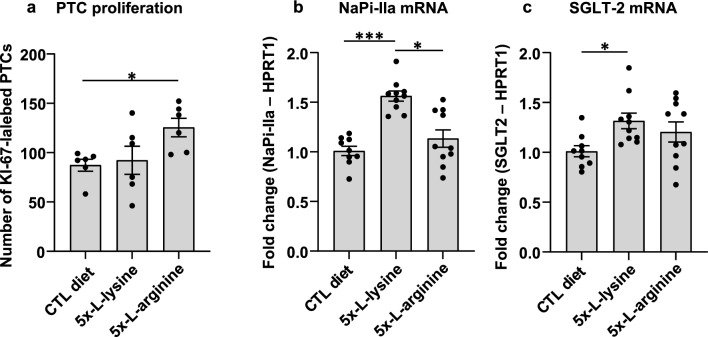
Effects of diets on selected PTC indices in *Ctns* KO mice kidneys at 8 months of age. (**a**) Proliferative events in PTCs were identified by Ki-67 immunolabelling in confocal images and scored over random fields to cumulative areas of 0.96 mm^2^. This data does not disclose protection against proliferation under 5x-l-lysine at this interval and rather indicates that 5x-l-arginine diet accelerates PTC turn-over in *Ctns* KO kidneys, suggesting higher PTC replacement after shedding (*P* < 0.05 by Kruskal–Wallis test). For representative confocal images and proliferation events in all cortical compartments, see Supplemental Fig. 6. (**b**,**c**) Apical differentiation state. Expression levels of NaPi-IIa and SGLT-2 as representative apical PT transporters were calculated as ratios to the housekeeping gene, HPRT1, and presented as fold-change viz control diet. Increased mRNA expression of both transporters suggests that 5x-l-lysine may offer partial protection against apical PT dedifferentiation in this animal model.

At cellular level, we assessed PT cell death linked to compensatory renewal, by measuring Ki-67 immunolabelling abundance, a validated proliferation index (Fig. 6a)^22^. This index was undistinguishable between control diet and 5x-l-lysine, but increased by 144% of control under 5x-l-arginine (*P* < 0.05), pointing to moderate toxicity by the 5x-l-arginine diet. At subcellular level, NaPi-IIa and SGLT-2 mRNA levels were measured as apical differentiation markers (Fig. 6b,c). This analysis suggested a significant yet limited benefit for both under 5x-l-lysine, compatible with modest functional cystinotic PTC protection.

## Discussion and perspectives

As first conclusion, free lysine and arginine can directly compete for megalin:cargo interaction at supraphysiological concentrations, as Biacore assays demonstrate. Inhibition is dose-dependent with no difference between l- and d-dibasic AA stereoisomers, supporting the original prediction of competition for binding to luminal surface “sites” of proximal kidney tubules by pairs of positive charges at appropriate spacing, lysine being strongest^10^. Our acellular competition study points to the importance of lysine/arginine residues in cargoes for recognition by Ca^2+^-coordinated clusters of acidic residues in megalin^12^. In this discussion, we will mainly focus on 5x-l-lysine, then compare with 5x-d-lysine and 5x-l-arginine.

As second conclusion, prolonged ad libitum solid diet supplemented with 5x-l-lysine appears well-tolerated in WT and *Ctns* KO mice, reaches average urinary concentrations (~ 25 mM in *Ctns* KO mice) in the range causing significant competition for megalin:protein interaction in acellular assay, and results into sustained albeit limited LMWPuria. The immediate dose-dependent induction of LMWPuria by injected high doses of l-lysine in human volunteers predicted direct competition for luminal binding^10^. However, an alternative/additional interpretation stemmed from the observation that acute l-lysine gavage to rats caused redistribution of megalin and cubilin to the endocytically incompetent brush border^13^. Upon sustained supplementation by l-lysine in WT mice, we found no comparable redistribution of apical megalin, nor effect on its endocytic cargo trafficking in PTCs. Taken together, we conclude that inhibition of endocytosis in 5x-l-lysine-treated mice involves competition for megalin binding at the luminal surface of mice kidney PTCs.

Daily feeding pattern of mice is discontinuous, so that significant variations of urinary dAA levels, thus of competition for LMWP uptake are expected across the day. This interpretation is supported by suppression of TxRed-ovalbumin cortical signal by 5x-lysine diet during active feeding hours (Fig. 4), but not during sleeping hours, compatible with intermittent competition. Thus, although the impact of 5x-l-lysine on proteinuria in 24-h urine collections was rather modest (no change on albuminuria relative to control diet; sevenfold relative increase of CC16 in WT mice but only twofold relative increase of CC16 in *Ctns* KO mice), this diet clearly inhibited cortical endocytic uptake during feeding hours. Oscillating daily endocytosis inhibitory phases with less or not protected phases must therefore be taken into consideration to evaluate the significance of the cumulative ~ twofold decrease by 5x-l-lysine of kidney cystine accumulation in *Ctns* KO mice over the two-months treatment (from 6-to-8 months of age). In marked contrast, there was minimal PTC protection against dedifferentiation, as indicated by a moderately higher kidney gene expression for the apical transporters NaPi-IIa and SGLT2 than under control diet (Fig. 6b,c), combined with the observed tendency to lower phosphaturia and glycosuria (Table 1), and no histological protection at 8 months-of age (contrasting with prevention of swan-neck deformities by 5x-l-lysine from 2-to-6 months-of-age). Whereas kidney cystine is a convenient quantitative marker of NC, it is still unsettled whether it reflects a causal relation with disease progression^25,38^.

Distal cargo redistribution from S1 to S3, documented here in WT mice upon 5x-l-lysine during feeding hours, was previously demonstrated by autoradiography of injected ^125^I-beta_2_-microglobulin in *untreated Ctns* KO mice at 12 months of age, a more advanced stage : total kidney radioactivity was surprisingly well-preserved as compared to age-matched WT mice, indicating effective distal compensation^31^. Indeed, the endocytic machinery is significantly different between S1 and S3 segments in mice^39,40^, and cystinotic lesions at the age of *Ctns* KO mice studied here occur essentially in S1 segment^31^. The first PT segment would thus appear to be protected against cystine accumulation by 5x-l-lysine, at least in part, by redistribution of endocytic load to competent S3, with much less appearance of LMWP in final urine.

Besides surface megalin competition, intracellular effects of l-lysine, not expected for d-lysine, must be considered. PTCs are a key body compartment for lysine metabolism. This metabolism can be impaired in disease, e.g. the sodium-dependent rat hypertensive model where oral lysine supplementation corrects clinical, metabolic and structural defects, as well as, paradoxically, albuminuria^35^. In addition, lysosomal mobilization of cystine is a key modulator of mTORC-1. Alternatively, increased PTC loading by l-lysine likely impacts on mTORC-1^34^, possibly resulting into some favorable effects, including on vesicular trafficking^41^. Of note, only a minor proportion of cystine generated upon lysosomal proteolysis in cystinotic cells is actually retained in these cells^42^. Thus, any effect on apical vesicular recycling from lysosomes could have a major impact on retention level (for the potential role of Rab27a, see^43^). Conversely, whereas mTORC-1 pathway is defective in immortalized cystinotic cells^44,45^, its pharmacological inhibition appeared protective in cystinotic stem cells^46^ and even corrected lysosomal dysfunction and endocytosis defect in differentiated cystinotic PTCs^47^. Full understanding of mTORC1 pathway in cystinotic kidneys and its status under 5x-l-lysine would require further investigation.

The stereoisomer, 5x-d-lysine, cannot enter PTCs due to stereospecificity of the Na^+^-independent rBAT/b^0,+^AT complex^36^, thus should not impact on mTORC-1 in these cells. 5x-d-lysine caused a remarkable *suppression* of cystine accumulation in cystinotic kidneys, as expected from complete competition for megalin at its huge urinary level. Although additional effects on PTC function or some toxicity cannot be excluded, in view of increased diuresis and glycosuria, such effects would be selective in view of no or minimal urinary loss of major neutral AAs (glycine, alanine), reabsorbed by potent Na^+^-dependent transporter^48^. Further investigations on the behaviour of PTCs under 5x-d-lysine (or lower doses) are warranted. Whether the lack of effect of d-lysine on *Ctns* KO liver cystine accumulation, concentrated in Küpffer cells, reflects a different sensitivity to competition for their scavenger/LRP-1 receptor also deserves to be examined.

The similar benefit of 5x-l-arginine as 5x-l-lysine on kidney cystine accumulation in *Ctns* KO mice could be surprising in view of much lower urinary arginine concentration and lower intrinsic competition potential for megalin. One possible explanation is the unexpected increase of cystinotic PT cellular turn-over under l-arginine, revealed by increased KI67 immunolabelling. This result would indicate PTC replacement after increased apoptotic shedding events, causing discharge of lysosomes with cystine crystals into urine^22^. In contrast to 5x-l-lysine, 5x-l-arginine failed to protect gene expression of NaPi-IIa and SGLT-2 in *Ctns* KO mice.

The obvious final question as to whether dAAs diet could be beneficial to cystinotic patients deserve some final cautionary comments. Indeed, based on our recent preliminary results (not shown), the same 5x-l-lysine diet applied to a new cystinotic *rat* model^21^ also considerably decreased kidney cystine accumulation. However, this was unfortunately accompanied by a serious worsening of the Fanconi syndrome, which is better reproduced in the rat than in the mouse model. Whether l-lysine supplementation to cystinotic rats further enhances the deleterious activation mTORC-1 due to cystinosis in differentiated PTCs^47^ deserves to be tested. Additional studies including the possibility to prevent or slow down kidney damage by earlier implementation with l-lysine, l-arginine, or their combination, with or without cysteamine, the potential benefit of lower doses of d-lysine; as well as integrated view of mechanisms and consequences of dAA diet, are clearly needed before considering testing dAA diet in cystinotic patients.

## Materials and methods

### Surface plasmon resonance

Surface plasmon resonance (SPR) analysis of the binding to chip-immobilized megalin of receptor-associated protein (RAP), transcobalamine:cobalamine complex (TC:B12) and lysozyme was carried out using a Biacore T200 instrument (Uppsala, Sweden). The sensor chips (type CM5) were activated with a 1:1 mixture of 0.2 M N-ethyl-N′-(3-dimethylaminopropyl) carbodiimide and 0.05 M N-hydroxysuccinimide in H_2_O. Porcine megalin^49^ was immobilized on the chip in 10 mM sodium acetate, pH 4.0, and the remaining binding sites were blocked with 1 M ethanolamine, pH 8.5. The SPR signal generated from immobilized megalin were obtained with a density of 4.5 fmol protein/mm^2^.

Human RAP was produced as recombinant protein in *E. coli*^50^ and used to validate the sensor chip. TC was purified and complexed with B12 as described (TC/B12)^50,51^. Human recombinant lysozyme was purchased from Sigma-Aldrich (expressed in rice, # L1667). Sensorgrams were generated using each of these three ligands at 10 µg/ml in a running buffer consisting of 10 mM Hepes, 150 mM NaCl, 4 mM CaCl_2_ and 0.05% Tween 20, pH 7.4. The flow cells were regenerated with 1 M ethanolamine pH 9.0. Analyte testing was performed as a dual injection starting with running buffer supplemented with the defined concentrations of amino acids followed by injection of analyte in the same solution. The pH of all buffers was adjusted after amino acids addition. All binding experiments were made as at least independent triplicate of technical duplicates and data were analyzed using the Biacore T200 evaluation software version 3.1.

### Mouse breeding and diet

*Ctns* knockout mice (C57BL/6 background) were kindly provided by Dr. Corinne Antignac^18^. Animal care and experimental procedures were conducted in accordance with the European 2010/63/EU directive on the protection of animals used for scientific purposes, followed the recommendations of the ARRIVE guidelines, and were authorized by the Italian Ministry of Health (authorization number 303/2020-PR). Except when stated otherwise, female wild type and *Ctns*^*−/−*^ mice were fed from 6-months of age with a control diet (4RF21 diet, Mucedola Srl, Settimo Milanese, Italy; containing 9.7 g l-lysine and 10.9 g l-arginine per kg of food) or with the same diet supplemented by 37.7 g of l-lysine (DBA Cat# GM5722) or 37.7 g of d-lysine (DBA Cat# GM3691) or 44.9 g of l-arginine (DBA Cat# GM7438) per kg of food, for 2 months. Based on our observations that mice with an average body weight of 22 g eat on average 3 g of food per day, the estimated corresponding doses in supplemented mice were 6.5 g/kg/day for l-lysine and d-lysine and 7.6 g/kg/day for l-arginine (or approx. 45 mmoles/kg/day for either compound). A fivefold increase of l-arginine in a chronic diet is considered safe in humans^52^. The same increase was set for l- (and d-) lysine. Whereas all these diets were well-tolerated, prolonged 5x-d-arginine caused weight loss and eventual high mortality (not shown), thus this arm was discontinued.

### Clinical serum and urine assays

After 24-h adaptation in metabolic cages, urine was collected during the following 24 h in presence of 0.1% sodium azide and 1x-protease inhibitors (Thermo Scientific). When in metabolic cages, mice had free access to tap water and food. Blood was collected 2 days before starting the treatments and entry into metabolic cages (baseline) and at the sacrifice to obtain serum. Body weight was recorded before entering the metabolic cages. Urine and serum analyses were performed by the veterinary laboratory Appialab Srl (Rome, Italy). Urinary levels of CC16 were measured by the Mouse Clara Cell Protein 16 kit 96 T #EKU03200 (Biomatik) according to manufacturer’s instructions. Serum levels of cystatin-C were measured by the Mouse/Rat Cystatin C Quantitative ELISA #MSCTC0 (R&D Systems). Creatinine clearance was calculated by the formula: creatinine clearance (mL/min) = urine creatinine (mg/dL) × 24 h-urine volume (mL)/serum creatinine (mg/dL) × 1440 min. Creatinine clearance was normalized by mouse body weight.

### Amino-acid concentration assay by mass spectrometry

Quantitative analysis of underivatized amino-acids in serum and urine samples was performed using the Amino Acids LC–MS/MS analysis Kit Jasem®. Liquid chromatography and mass spectrometry analysis was performed by a UHPLC Agilent 1290 Infinity II 6470 (Agilent Technologies) equipped with an ESI-JET-STREAM source operating in the positive ion (ESI+) mode. Equipment was controlled and data were analyzed using MassHunter Workstation (Agilent Technologies). The assay was validated for selectivity, specificity, linearity and limit of quantification, accuracy and precision, matrix effects and recovery, and stability.

### Tissue cystine assay

Mice were euthanized by cabon dioxide (CO2) inhalation. Dissected tissues were homogenized in 10 mM N-ethylmaleimide. Protein fraction was obtained after precipitation in 10% 5-sulfosalicylic acid and centrifugation at 20,000*g* for 20 min at 4 °C. Protein pellet was resuspended in 100 mM NaOH and assayed by the Bio-Rad Protein Assay according to manufacturer’s instructions. Supernatant (50 μl) was mixed with 50 μL of the internal standard solution (Cystine d6) and vortexed for 5 s; then the mixture was extracted with 200 μl of acetonitrile, vortexed for at least 30 s, then centrifuged at 13,000 rpm for 9 min. Liquid chromatography and mass spectrometry analysis was performed by a UHPLC Agilent 1290 Infinity II 6470 (Agilent Technologies) equipped with an ESI-JET-STREAM source operating in the positive ion (ESI +) mode. Equipment was controlled and data were analyzed using MassHunter Workstation (Agilent Technologies). The separation column was InfinityLab Poroshell 120 HILIC 1.9 μm 100 × 2.1 mm (Agilent Technologies). Validation of the method was performed basing on the US Food and Drug Administration (FDA) guideline for industry bioanalytical method validation (FDA 2013) and European Medicines Agency (EMA) guideline (EMA 2011). The assay was validated for selectivity, specificity, linearity and limit of quantification, accuracy and precision, matrix effects and recovery, and stability.

### Confocal fluorescence imaging and Sirius Red staining

For in vivo endocytic tracing in PTCs, Texas Red-ovalbumin (300 µg, Invitrogen #O23021) was injected intravenously at 20 min before sacrifice. For immunofluorescence, antigen retrieval was promoted in citrate buffer, pH 6, at 95 °C for 20 min using a Lab Vision Pretreatment Module™ (Thermo Scientific). Tissue was permeabilized with PBS/0.3% Triton-X100 for 5 min, then for one further hour with 10% bovine serum albumin (BSA)/3% milk to block non-specific sites. Sections were incubated overnight at 4 °C with the following primary reagents in blocking buffer: sheep anti-megalin (1/800, kindly provided by Drs P. Verroust and R. Kozyraki, INSERM U968, Paris, F-75012, FR), rat anti-LAMP-1 (1/100; #1D4B, Hybridoma Bank), mouse anti-ezrin (1/100, #MS-661-P1, ThermoScientific), mouse anti-Ki67 (1/250, #556003, BD-Pharmingen), rabbit anti-human albumin (1/1000; Dako #A0001). After washing, sections were further incubated with the appropriate AlexaFluor-secondary antibodies for 1 h at room temperature in 10% BSA/0.3% Triton-X100 and mounted with Faramount aqueous mounting medium (Dako). Sections were imaged on a spinning disk confocal microscope using EC Plan-NeoFluar 40X/1.3 or 100x/1.4 Oil DIC objective (Cell Observer Spinning Disk; Zeiss). Proliferative events in kidney cortex were quantified as described^22^. Alternatively, whole kidney images were acquired using a Pannoramic 250 Flash III microscope (3DHistech). Histological PAS staining and quantification of the frequency of swan-neck lesions were performed as described^22^. Sirius Red staining of paraffine sections and morphometry in total sagittal kidney sections after conversion to red fluorescence were performed as described^53,54^**.**

### Statistical analyses

Except at dot plots, values are expressed as mean ± SEM of the indicated numbers. Statistical analyses were performed by non-parametric tests. Significance of comparison between two groups only was assessed by Mann–Whitney tests. For multiple group comparison, differences among groups were assessed by the non-parametric Kruskal–Wallis test. If differences were significant, pairwise comparisons between each treated group and control group were evaluated by Dunn’s multiple comparison test. *P*-values < 0.05 were considered significant and denoted *; ** for *P* < 0.01; *** for *P* < 0.001; and **** for *P* < 0.0001.

### Supplementary Information

Supplementary Figures.

## Data Availability

Materials, data and associated protocols are available by the corresponding author.

## References

[1] Nielsen R, Christensen EI, Birn H (2016). Megalin and cubilin in proximal tubule protein reabsorption: From experimental models to human disease. Kidney Int..

[2] Perez Bay AE (2016). The fast-recycling receptor Megalin defines the apical recycling pathway of epithelial cells. Nat. Commun..

[3] Shipman KE (2022). An adaptable physiological model of endocytic megalin trafficking in opossum kidney cells and mouse kidney proximal tubule. Function.

[4] Grieco G (2018). Vps34/PI3KC3 deletion in kidney proximal tubules impairs apical trafficking and blocks autophagic flux, causing a Fanconi-like syndrome and renal insufficiency. Sci. Rep..

[5] Rinschen MM (2022). VPS34-dependent control of apical membrane function of proximal tubule cells and nutrient recovery by the kidney. Sci. Signal..

[6] Christensen EI, Wagner CA, Kaissling B (2012). Uriniferous tubule: Structural and functional organization. Compr. Physiol..

[7] Nielsen R (2007). Endocytosis provides a major alternative pathway for lysosomal biogenesis in kidney proximal tubular cells. Proc. Natl. Acad. Sci. USA.

[8] Johanns M (2017). Cellular uptake of proMMP-2:TIMP-2 complexes by the endocytic receptor megalin/LRP-2. Sci. Rep..

[9] Moestrup SK (1995). Evidence that epithelial glycoprotein 330/megalin mediates uptake of polybasic drugs. J. Clin. Investig..

[10] Mogensen CE, Sølling K (1977). Studies on renal tubular protein reabsorption: Partial and near complete inhibition by certain amino acids. Scand. J. Clin. Lab. Investig..

[11] Ottosen PD (1985). Inhibition of protein reabsorption in the renal proximal tubule by basic amino acids. Ren. Physiol..

[12] Andersen CB, Moestrup SK (2014). How calcium makes endocytic receptors attractive. Trends Biochem. Sci..

[13] Thelle K (2006). Characterization of proteinuria and tubular protein uptake in a new model of oral l-lysine administration in rats. Kidney Int..

[14] Jamar F (2003). 86Y-DOTA0)-D-Phe1-Tyr3-octreotide (SMT487)–a phase 1 clinical study: Pharmacokinetics, biodistribution and renal protective effect of different regimens of amino acid co-infusion. Eur. J. Nucl. Med. Mol. Imaging.

[15] Barone R (2005). Endocytosis of the somatostatin analogue, octreotide, by the proximal tubule-derived opossum kidney (OK) cell line. Kidney Int..

[16] Molema F (2019). Evaluation of dietary treatment and amino acid supplementation in organic acidurias and urea-cycle disorders: On the basis of information from a European multicenter registry. J. Inherit. Metab. Dis..

[17] Elpeleg N, Korman SH (2001). Sustained oral lysine supplementation in ornithine delta-aminotransferase deficiency. J. Inherit. Metab. Dis..

[18] Nevo N (2010). Renal phenotype of the cystinosis mouse model is dependent upon genetic background. Nephrol. Dial Transplant..

[19] Cheung PY (2021). In vitro and in vivo models to study nephropathic cystinosis. Cells.

[20] Hollywood JA (2022). Cystinosin-deficient rats recapitulate the phenotype of nephropathic cystinosis. Am. J. Physiol. Renal Physiol..

[21] Krohn P (2022). Multisystem involvement, defective lysosomes and impaired autophagy in a novel rat model of nephropathic cystinosis. Hum. Mol. Genet..

[22] Janssens V (2019). Protection of cystinotic mice by kidney-specific megalin ablation supports an endocytosis-based mechanism for nephropathic cystinosis progression. J. Am. Soc. Nephrol..

[23] Cherqui S (2001). The targeting of cystinosin to the lysosomal membrane requires a tyrosine-based signal and a novel sorting motif. J. Biol. Chem..

[24] Kalatzis V (2001). Cystinosin, the protein defective in cystinosis, is a H(+)-driven lysosomal cystine transporter. EMBO J..

[25] Gahl WA, Thoene JG, Valle DL (2019). Cystinosis: A disorder of lysosomal membrane transport. The Online Metabolic and Molecular Bases of Inherited Disease.

[26] Cherqui S, Courtoy PJ (2017). The renal Fanconi syndrome in cystinosis: Pathogenic insights and therapeutic perspectives. Nat. Rev. Nephrol..

[27] Adelmann CH (2020). MFSD12 mediates the import of cysteine into melanosomes and lysosomes. Nature.

[28] Thoene JG, Lemons RM (1982). Cystine accumulation in cystinotic fibroblasts from free and protein-linked cystine but not cysteine. Biochem. J..

[29] Lloyd JB (1986). Disulphide reduction in lysosomes. The role of cysteine. Biochem. J..

[30] Jamalpoor A (2021). molecular mechanisms and treatment options of nephropathic cystinosis. Trends Mol. Med..

[31] Gaide Chevronnay HP (2014). Time course of pathogenic and adaptation mechanisms in cystinotic mouse kidneys. J. Am. Soc. Nephrol..

[32] Raggi C (2014). Dedifferentiation and aberrations of the endolysosomal compartment characterize the early stage of nephropathic cystinosis. Hum. Mol. Genet..

[33] Ivanova EA (2015). Endo-lysosomal dysfunction in human proximal tubular epithelial cells deficient for lysosomal cystine transporter cystinosin. PLoS ONE.

[34] Fotiadis D, Kanai Y, Palacín M (2013). The SLC3 and SLC7 families of amino acid transporters. Mol. Aspects Med..

[35] Rinschen MM (2022). Accelerated lysine metabolism conveys kidney protection in salt-sensitive hypertension. Nat. Commun..

[36] Mizoguchi K (2001). Human cystinuria-related transporter: Localization and functional characterization. Kidney Int..

[37] Boger RH, Bode-Boger SM (2001). The clinical pharmacology of l-arginine. Annu. Rev. Pharmacol. Toxicol..

[38] Prencipe G (2014). Inflammasome activation by cystine crystals: Implications for the pathogenesis of cystinosis. J. Am. Soc. Nephrol..

[39] Jouret F (2007). Cystic fibrosis is associated with a defect in apical receptor-mediated endocytosis in mouse and human kidney. J. Am. Soc. Nephrol..

[40] Schuh CD (2018). Combined structural and functional imaging of the kidney reveals major axial differences in proximal tubule endocytosis. J. Am. Soc. Nephrol..

[41] Grahammer F (2017). mTOR regulates endocytosis and nutrient transport in proximal tubular cells. J. Am. Soc. Nephrol..

[42] Thoene JG, Lemons R (1980). Modulation of the intracellular cystine content of cystinotic fibroblasts by extracellular albumin. Pediatr. Res..

[43] Johnson JL (2013). Upregulation of the Rab27a-dependent trafficking and secretory mechanisms improves lysosomal transport, alleviates endoplasmic reticulum stress, and reduces lysosome overload in cystinosis. Mol. Cell Biol..

[44] Andrzejewska Z (2016). Cystinosin is a component of the vacuolar H+-ATPase-ragulator-rag complex controlling mammalian target of rapamycin complex 1 signaling. J. Am. Soc. Nephrol..

[45] Ivanova EA (2016). Altered mTOR signalling in nephropathic cystinosis. J. Inherit. Metab. Dis..

[46] Hollywood JA (2020). Use of human induced pluripotent stem cells and kidney organoids to develop a cysteamine/mTOR inhibition combination therapy for cystinosis. J. Am. Soc. Nephrol..

[47] Berquez, M. *et al.* Lysosomal cystine export regulates mTORC1 signaling to guide kidney epithelial cell fate specialization. *bioRxiv* 2022.08.28.505580 (2022). 10.1038/s41467-023-39261-3 PMC10349091 37452023

[48] Christensen HN (1990). Role of amino acid transport and countertransport in nutrition and metabolism. Physiol. Rev..

[49] Moestrup SK (1993). Epithelial glycoprotein-330 mediates endocytosis of plasminogen activator-plasminogen activator inhibitor type-1 complexes. J. Biol. Chem..

[50] Moestrup SK (1996). Megalin-mediated endocytosis of transcobalamin-vitamin-B12 complexes suggests a role of the receptor in vitamin-B12 homeostasis. Proc. Natl. Acad. Sci. USA.

[51] Hansen M, Nexø E (1992). Cobalamin binding proteins in human seminal plasma. Scand. J. Clin. Lab. Investig..

[52] McNeal CJ (2016). Safety and effectiveness of arginine in adults. J. Nutr..

[53] Courtoy GE (2020). Digital image analysis of picrosirius red staining: A robust method for multi-organ fibrosis quantification and characterization. Biomolecules.

[54] Vogel B (2015). Determination of collagen content within picrosirius red stained paraffin-embedded tissue sections using fluorescence microscopy. MethodsX.

