# Supplementary material for: Dietary supplementation of cystinotic mice by lysine inhibits the megalin pathway and decreases kidney cystine content
Source: Sci Rep. 2023 Oct 12;13:17276. doi: 10.1038/s41598-023-43105-x (PMC10570359; doi:10.1038/s41598-023-43105-x)
Supplement: Supplementary file 1 — Supplementary Figures. [file 41598_2023_43105_MOESM1_ESM.pdf]

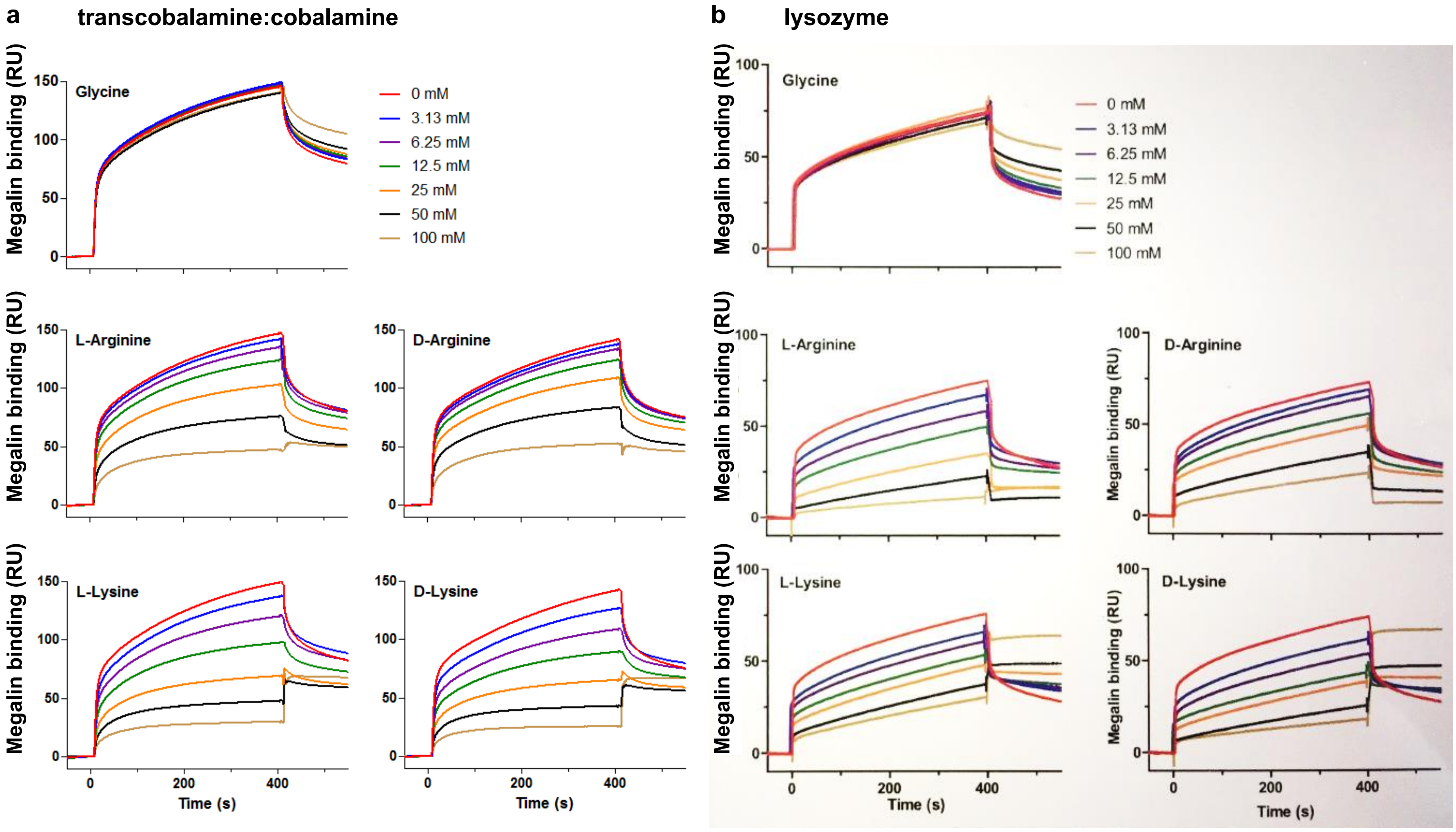

Supplemental Figure 1

Supplemental Figure 1 *(complementary to Figure 1)*. **Primary sensorgrams** testing the effects of the indicated amino-acids on the binding to immobilized megalin of transcobalamine/cobalamine complex at left (as plotted in Figure 1) or lysozyme, at right. RU, relative units. Notice pattern similarities between the two megalin cargoes and between L- *vs* D-AAs.

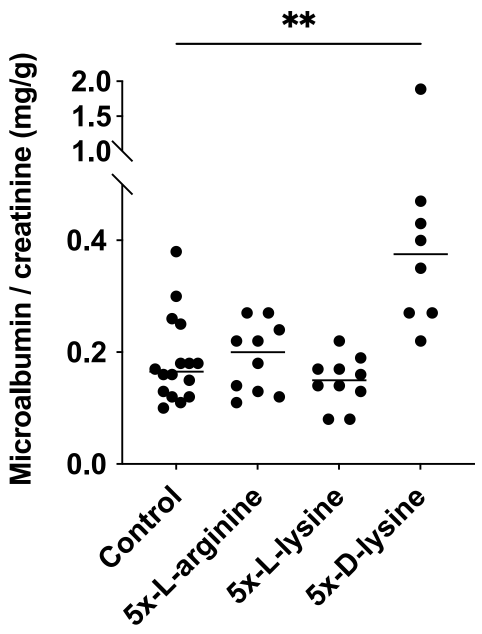

Supplemental Figure 2 *(complementary to Table I)*

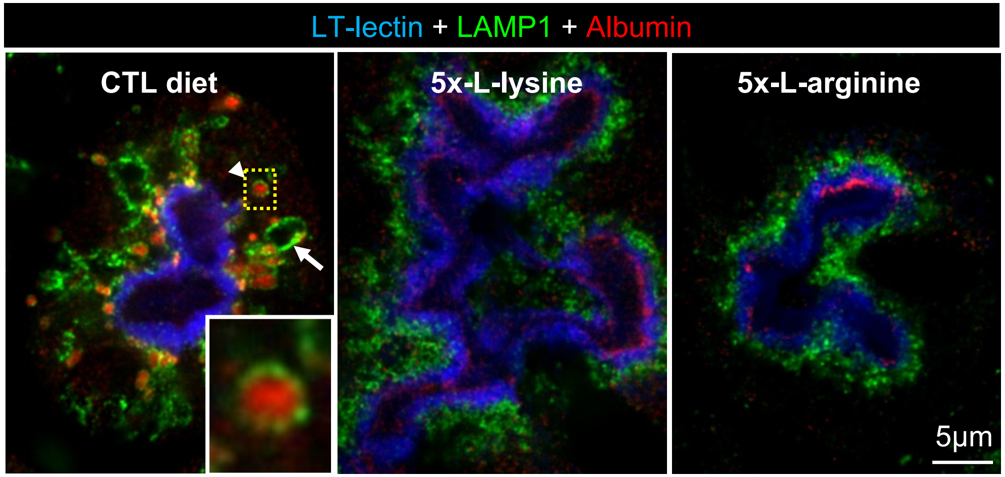

Supplemental Figure 3.

Supplemental Figure 3. Effect of primary treatment by dAA diets in *Ctns* KO female mice from 2-to-6 months-of-age: prevention of lysosomal enlargement/deformation and loss of detection of endogenous albumin labeling. At 2 months of age, mice were either left untreated (CTL diet) or treated for 4 months by oral supplementation with 5x-L-lysine or 5x-L-arginine. At 6 months-of-age, kidneys were perfusion-fixed and processed for triple fluorescence for brush border (*Lotus Tetragonolobus* lectin, LT-lectin, blue), lysosomes (LAMP1, green) and endogenous mouse albumin (red). Untreated cystinotic PTCs frequently bear enlarged, distorted lysosomes (white arrow, compatible with large cystine crystal) with accumulating albumin (white arrowhead, enlarged below: red albumin disk encircled by green LAMP1). In contrast, both 5x- L-lysine and 5x-L-arginine appear to prevent lysosomal enlargement and distorsion. In addition, albumin is not detected within PT lysosomes of dAA-treated mice under identical immunolabeling/imaging conditions, indicating impaired endocytosis and/or preserved degradation upon dbAA supplementation. Inconstant red signal at brush border may represent fixation artefact due to postmortem cross-linking of luminal albumin. Scale bar, 5 µm.

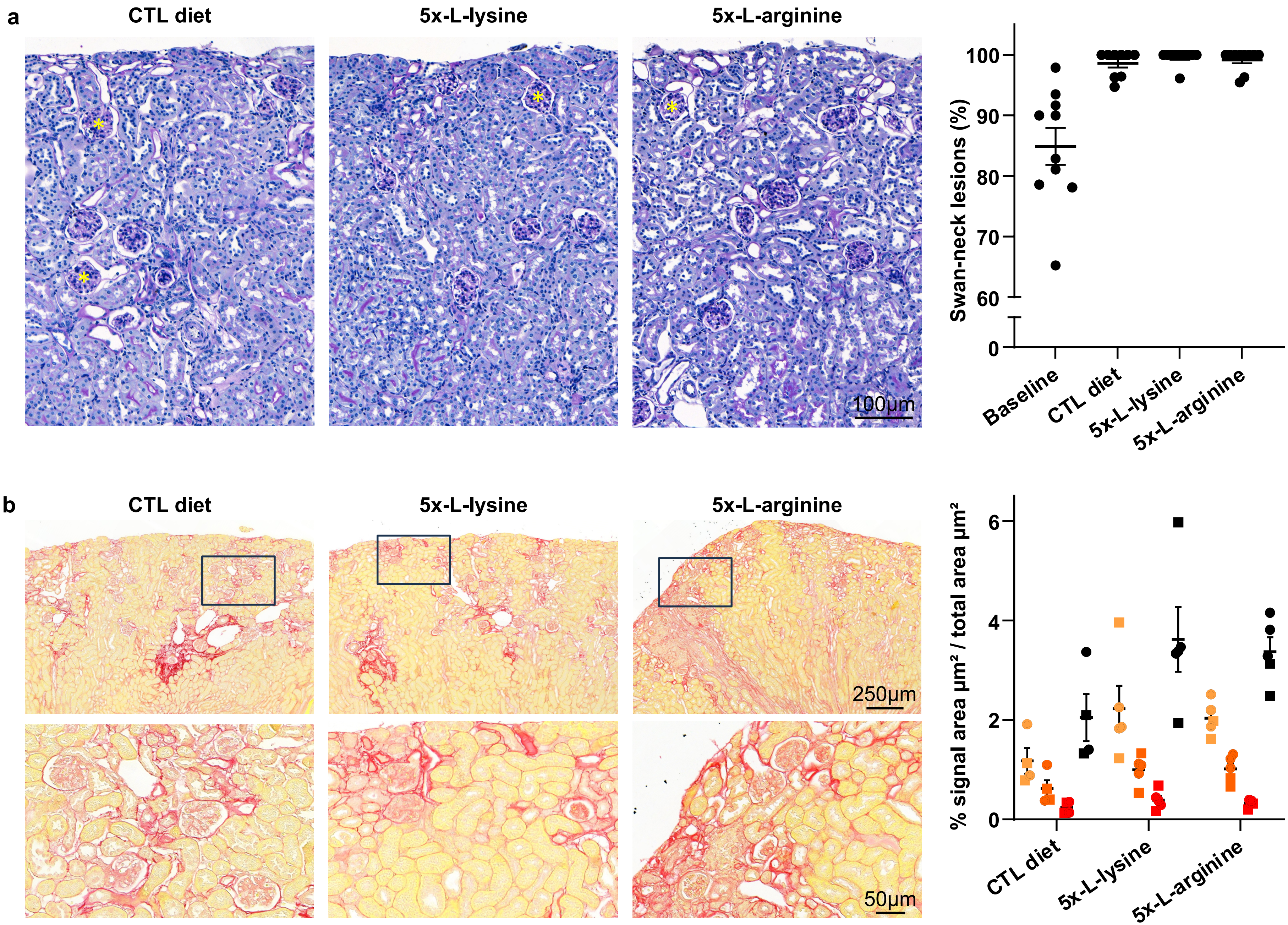

Supplemental Figure 4

Supplemental Figure 4. Histological survey with focus on swan-necks and assessment of fibrosis in *Ctns* KO mice treated with dAA diets from 6-to-8 months-of-age. (a,b) PAS staining. (a) Representative images of kidney cortex from *Ctns* KO female mice at 8 months-of-age stained by PAS at intermediate magnification (scale bar, 100 µm). Yellow asterisks denote typical swan-neck lesions visible in this plane of section. No obvious diet effect is seen. **(b)** Quantification of swan-neck frequency at 6 months of age (baseline) and at 8 months of age under control, 5x-L-lysine or 5x-L-arginine diets. For the significance of swan-necks, please see [26]. For more information on swan-neck quantification, see [22]. No protection is seen in this cohort. However, for a substantial protective effect of L-lysine by primary prevention (2-to-6 months-of-age), see Supplemental Figure 5. **(c,d) Sirius red staining**. **(c)** Representative images of kidney cortex for Ctns KO female mice at 8 months-of-age or males at 9 month-of-age (to match slower disease progression in males) under control, 5x-L-lysine or 5x-L-arginine diets at low (scale bar, 250 µm) and high magnification (scale bar, 50 µm). Fibrotic areas are colored in red. No obvious diet effect is seen. **(d)** Whole kidney cortex quantification of the percentage of fibrotic area based on Sirius Red-induced fluorescence in females (filled circles) and males (at 9 month-of-age to match slower disease progression in males, filled squares) using HALO software to stratify three fluorescence intensity levels (weak, yellow; moderate, orange; strong, red; total signal in black). No protection by dAA diets is seen.

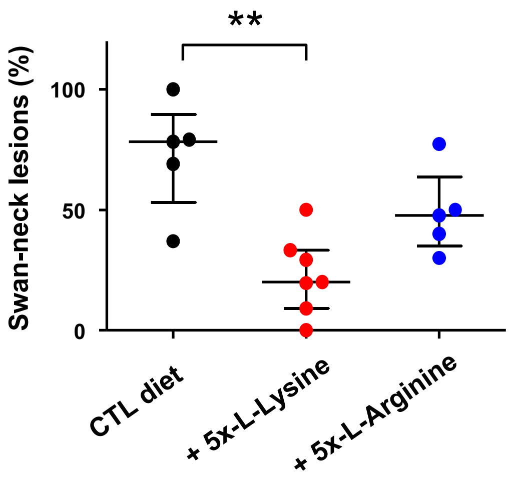

Supplemental Figure 5

Supplemental Figure 5 (complementary to Supplemental Figure 4). **Primary protection of Ctns KO female mice by dAA diets from 2-to-6 months-of-age decreases swan-neck deformities**. **, P<0.01 by non-parametric tests.

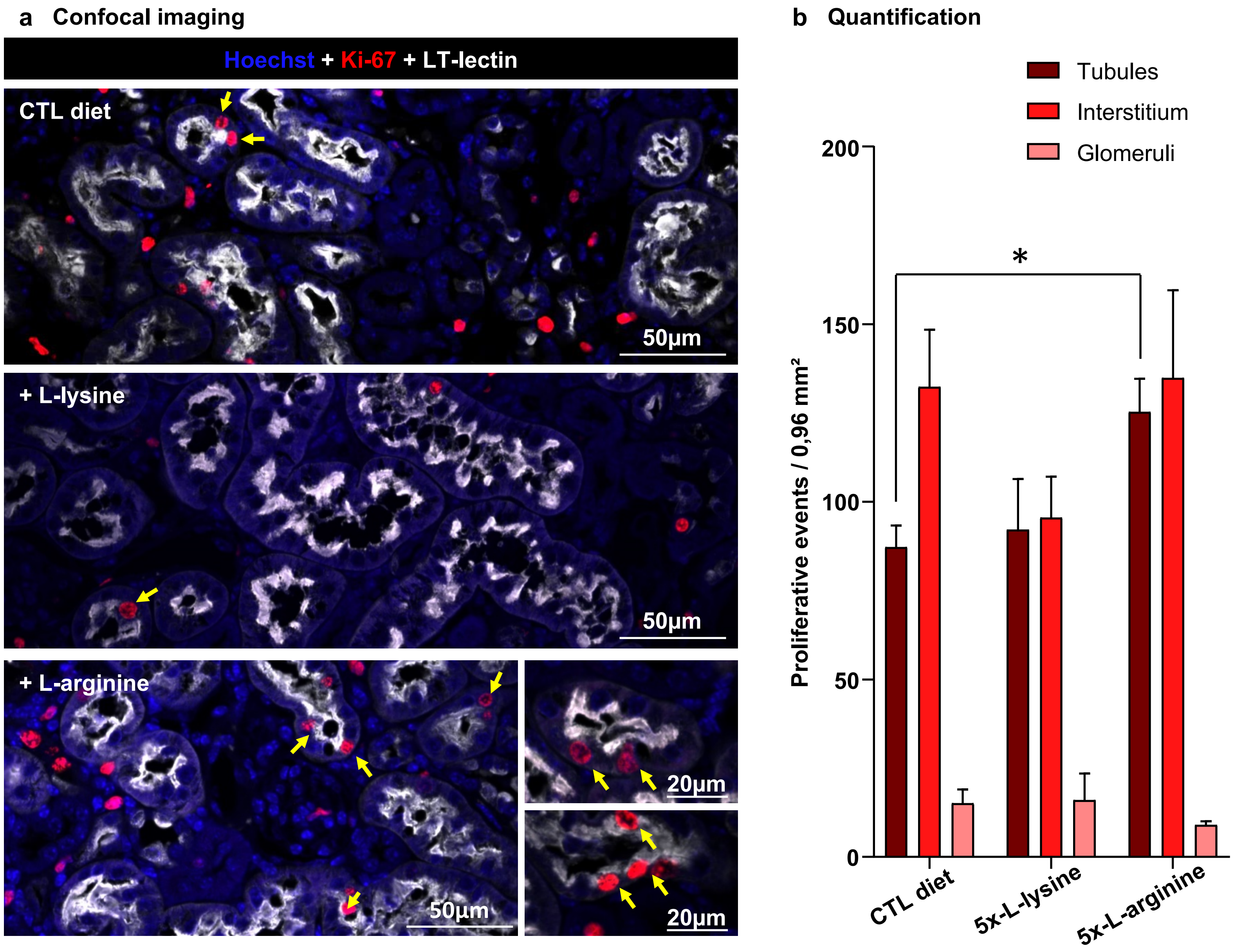

Supplemental Figure 6

Supplemental Figure 6 (complementary to Figure 6a). Assessment of cell proliferation in Ctns KO kidneys at 8 months-of-age. (**a**) Paraffine sections were triple-labeled for nuclei (Hoechst, blue), proliferation events (Ki-67 immunolabelling, red) and PT apex (direct *Lotus Tetragonolobus* lectin labeling, shown here in white) and analysed by confocal microscopy. For details of methods, see [22] ; for details of quantification (**b**), see Legend to Fig 6A. Yellow arrows point to KI-67-labeled PT nuclei. This data suggests that 5x-L-arginine selectively accelerates PT turn-over (*P*<0.05). The tendency towards lesser proliferation in interstitial cells under 5x-L-lysine, albeit interesting in the context of an inflammatory disease (Lobry et al, KI, 2019), is not significant.
